# Programming of Stress-Sensitive Neurons and Circuits by Early-Life Experiences

**DOI:** 10.3389/fnbeh.2019.00030

**Published:** 2019-02-18

**Authors:** Jessica L. Bolton, Annabel Katherine Short, Kristina A. Simeone, Jennifer Daglian, Tallie Z. Baram

**Affiliations:** Departments of Pediatrics, Anatomy/Neurobiology, Neurology, University of California, Irvine, Irvine, CA, United States

**Keywords:** CRH (corticotropin-releasing hormone), circuits, NRSF (neuron-restrictive silencer factor), limited bedding and nesting, brown adipose tissue, anhedonia

## Abstract

Early-life experiences influence brain structure and function long-term, contributing to resilience or vulnerability to stress and stress-related disorders. Therefore, understanding the mechanisms by which early-life experiences program specific brain cells and circuits to shape life-long cognitive and emotional functions is crucial. We identify the population of corticotropin-releasing hormone (CRH)-expressing neurons in the hypothalamic paraventricular nucleus (PVN) as a key, early target of early-life experiences. Adverse experiences increase excitatory neurotransmission onto PVN CRH cells, whereas optimal experiences, such as augmented and predictable maternal care, reduce the number and function of glutamatergic inputs onto this cell population. Altered synaptic neurotransmission is sufficient to initiate large-scale, enduring epigenetic re-programming within CRH-expressing neurons, associated with stress resilience and additional cognitive and emotional outcomes. Thus, the mechanisms by which early-life experiences influence the brain provide tractable targets for intervention.

## Introduction

The origins of mental health outcomes are early in life (Kessler et al., 2005; Insel, 2009). Genetic factors and early-life experiences, both adverse and nurturing, interact to modulate vulnerability and resilience to disease (Insel, 2009; Bale et al., 2010; Fox et al., 2010; Gunnar, 2010; Juul et al., 2011; Bale, 2015). There is now a large body of evidence in humans associating early-life adversity with emotional and cognitive disorders later in life (e.g., Bremner et al., 1993; Brown et al., 1995; Kaplan et al., 2001; Eriksson et al., 2014). To elucidate causal and mechanistic relationships between early-life experiences and later mental health, nonhuman primate (Koch et al., 2014; Wakeford et al., 2018) and rodent models have been utilized, specifically paradigms of adverse vs. optimal early-life experiences, which provoke cognitive and emotional changes relevant to human health (Weaver et al., 2004; Molet et al., 2014; Chen and Baram, 2016; Krugers et al., 2017; Walker et al., 2017; van Bodegom et al., 2017). Here, we discuss these animal models and provide new information on two major categories of changes in brain development that underlie risk vs. resilience to mental disorders: (1) circuit development; and (2) epigenetic alterations, as well as their mechanisms.

## Early-Life Adversity

**Figure 1:**
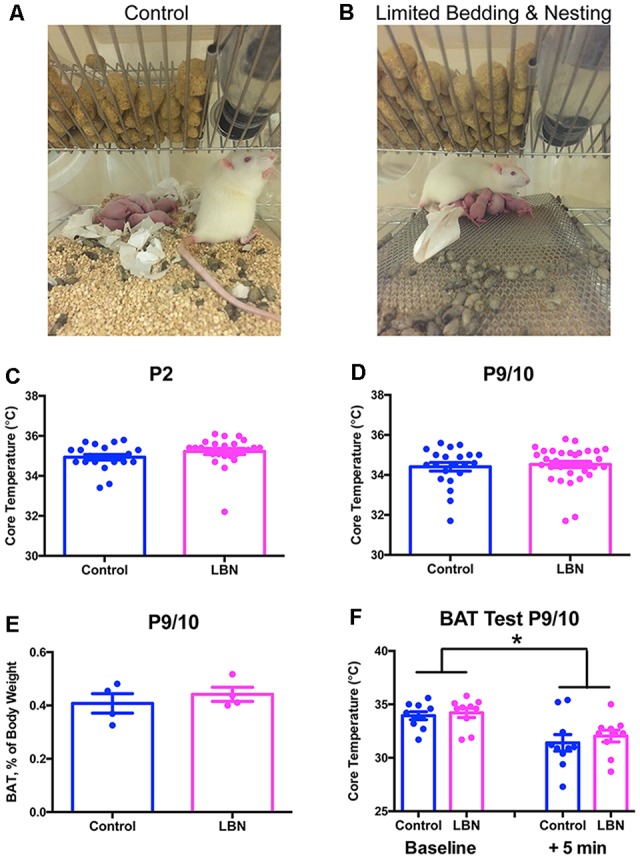
Limited bedding and nesting do not induce hypothermia or alter brown adipose tissue (BAT) thermogenesis. **(A,B)** Photographs of standard cages (Control; **A**) and limited bedding and nesting (LBN) cages **(B)**. **(C,D)** Core (rectal) temperatures do not differ at the beginning of the LBN experience on P2 **(C)** or at the end of the LBN experience on P9/10 (**D**; *t*-test, *p* > 0.1). Data are mean ± SEM; *n* = 21–33/group. **(E)** Gross wet weights of BAT (subscapular depot) do not differ between groups, regardless of whether data are normalized by body weight (*t*-test, *p >* 0.1). Data here are normalized to body weight mean ± SEM; *n* = 4/group. **(F)** Core temperatures do not differ by caging condition at baseline on P9/10, or 5 min after the initiation of cold stress. Cold stress consisted of 5 min at 4°C, followed by immediate measurement of core temperature. Data are mean ± SEM; *n* = 10/group. No significant between-group differences by repeated-measures two-way ANOVA; *p* > 0.1; significant within-subjects effect of time, *F*_(1,18)_ = 11.01, *p < 0.005. Note that data from male and female pups are combined here due to an absence of sex differences.

Experimental model studies, together with human studies, have found that maternal input has perhaps the most significant influence on the environment experienced during development (Bowlby, 1950; Seay et al., 1962; Baram et al., 2012; Rincón-Cortés and Sullivan, 2014; Kundakovic and Champagne, 2015). Thus, most animal models of early-life adversity have manipulated maternal interaction with pups, disrupting either the quantity or quality of maternal care early in life (for recent reviews see Molet et al., 2014; Walker et al., 2017; van Bodegom et al., 2017). This typically involves maternal separation or simulated poverty; in this perspective we shall focus on the latter. To simulate poverty, nesting and bedding materials in the cage are restricted (limited bedding and nesting: LBN) during postnatal days (P)2–9 (Figures 1A,B; Gilles et al., 1996; Molet et al., 2014; Naninck et al., 2014). This manipulation reliably and reproducibly causes fragmented and unpredictable maternal behaviors towards the pups (Molet et al., 2016a). Notably, the overall duration or quality of the nurturing behaviors of the dams are minimally altered; it is the patterns of maternal care that are disrupted (Ivy et al., 2008; Rice et al., 2008; Molet et al., 2016a; Walker et al., 2017). This fragmented maternal care causes chronic, unpredictable and uncontrollable “emotional stress” in the pups (Gilles et al., 1996; Ivy et al., 2008; Rice et al., 2008; Moriceau et al., 2009; Wang et al., 2011; Molet et al., 2014; Naninck et al., 2014). The stress is apparent in persistent elevations of plasma corticosterone and adrenal hypertrophy, and is associated with emotional and cognitive problems in adulthood (Brunson et al., 2005; Rice et al., 2008; Molet et al., 2016b). Notably, children that experience early-life adversity have an increased risk of developing similar difficulties, such as memory deficits, self-control issues, and internalizing disorders (Davis et al., 2017; Glynn et al., 2018).

### Circuit Development

Consistent with behavioral deficits, early-life adversity is associated with alterations in brain connectivity in humans, as apparent from neuroimaging studies (Posner et al., 2016; Fareri et al., 2017; Kopala-Sibley et al., 2018). Neuroimaging in rodents has identified increased structural connectivity between the medial prefrontal cortex and amygdala in adolescent LBN male rats, in association with anhedonia-like behavior (Bolton et al., 2018a). However, functional resting-state connectivity of amygdala to medial prefrontal cortex is diminished in LBN male rats pre-weaning and in adulthood. This was interpreted as suggesting that neonatal amygdala activation may accelerate the maturation of amygdala to prefrontal cortex projections and impact the functional integrity of such connections (Guadagno et al., 2018). Clearly, additional studies are required to discern how early-life adversity influences amygdala-cortical connectivity.

In experimental models, in addition to neuroimaging, one can assess directly synaptic connectivity via neuroanatomical studies. For example, early-life adversity causes increased excitatory connections onto corticotropin-releasing hormone (CRH)-expressing neurons in the paraventricular nucleus of the hypothalamus (PVH; Gunn et al., 2013). These neurons play a critical role in the stress response (Deussing and Chen, 2018), and thus their increased excitatory innervation may promote prolonged responses to stress in LBN animals (Gilles et al., 1996). Conversely, hippocampal regions exhibit reduced dendritic arborization and synaptic connections in LBN rats, which has been linked to adversity-induced memory deficits throughout life (Brunson et al., 2005; Ivy et al., 2010; Molet et al., 2016b). Thus, the effects of early-life adversity are brain region-dependent.

It is important to note that a specific stress-related consequence never stems exclusively from one single neuropeptide, but rather a combined interaction of many different neurotransmitter and/or neuropeptide systems. For example, oxytocin is a hypothalamic neuropeptide that has been implicated as a key molecule in the regulation of fear and anxiety, and has been shown to have effects on other neurotransmitter systems. Specifically, early-life adversity can impact oxytocin receptor expression of adult animals (Neumann and Slattery, 2016), and oxytocin has been shown to regulate CRH expression within the PVN (Jurek et al., 2015). Here, due to the limited scope of this perspective, we focus primarily on the programming of CRH neurons by early-life experiences.

### Epigenetic Alterations

Long-term phenotypic changes resulting from early-life adversity involve enduring alterations in the expression of important neuronal genes. A large body of work has now shown that early-life adversity is associated with transcriptional changes in both humans (Heim and Binder, 2012; Suderman et al., 2014; Schwaiger et al., 2016) and various rodent models (McClelland et al., 2011; Russo et al., 2012; Lucassen et al., 2013; Nestler, 2014; Szyf et al., 2016; Deussing and Jakovcevski, 2017; Peña et al., 2017; Ross et al., 2017; Gray et al., 2018). Much of the literature has focused on genes and gene families involved directly with stress, such as the glucocorticoid receptor (GR; Weaver et al., 2004; Gray et al., 2017). Early-life adversity decreases expression of GR in the PVN and frontal cortex in LBN rats (Avishai-Eliner et al., 2001), resulting in altered glucocorticoid negative feedback. In addition to GR, changes in Fkbp5 have been reported. Fkbp5 is a co-chaperone protein that binds to cytosolic GR in its inactive state, thus inhibiting GR translocation and function. Genetic polymorphisms of Fkbp5 in humans mediate a gene by environment interaction of childhood abuse and severity of PTSD symptoms later in life (Segman et al., 2005; Binder et al., 2008; Yehuda et al., 2009). Specifically, alleles associated with greater expression or function of Fkbp5, thought to result in GR hypersensitivity, are associated with greater vulnerability to PTSD symptoms after childhood abuse (Binder et al., 2008).

CRH is a key stress-related neuronal gene shown to be regulated by early-life adversity. Acutely after early-life adversity, CRH levels in the PVN are reduced in LBN rats and mice (Avishai-Eliner et al., 2001; Rice et al., 2008), likely as a result of depletion of CRH stores by the enhanced release of the peptide during the adversity period (Ma and Lightman, 1998). Over the past decade, the expression and function of CRH in brain regions outside the PVN has been established (Chen et al., 2001; Regev et al., 2011, 2012; Deussing and Chen, 2018). Notably, early-life adversity influences CRH expression in several of these regions. CRH is upregulated in the central nucleus of the amygdala (ACe; Dubé et al., 2015) and hippocampus of LBN rats (Brunson et al., 2001; Ivy et al., 2010). CRH is required for normal pruning and refinement of dendritic trees and synapses (Chen et al., 2004). In addition, excessive levels of CRH have been linked with aberrant neuronal structure and function (Chen et al., 2004; Ivy et al., 2010; Curran et al., 2017; Gunn and Baram, 2017).

Early adversity-induced changes in gene expression can be lifelong because of epigenetic processes involving master regulators, such as GR (Joëls and Baram, 2009; Schmidt et al., 2013; McEwen et al., 2016; Gray et al., 2017; van Bodegom et al., 2017). GR acts as a transcription factor along with several interacting proteins (Binder, 2009; Klengel and Binder, 2015; Xu et al., 2017; Ke et al., 2018) to modulate gene expression long-term and influence neuronal function (Daskalakis et al., 2015). Notably, recent mechanistic studies employing LBN (Arp et al., 2016; Lesuis et al., 2018) or maternal deprivation (Loi et al., 2017) have now directly confirmed a key role for GR in mediating the effects of early-life adversity on long-term brain function.

In addition to GR, our recent work has revealed an unexpected regulator of long-term changes in gene expression provoked by early-life adversity, the transcriptional repressor neuron-restrictive silencer factor (NRSF; Schulmann et al., 2018). This transcription factor has long been known to regulate CRH expression (Seth and Majzoub, 2001). Recently, RNA-seq detected transcriptome-wide changes in the dorsal hippocampus of LBN rats; surprisingly, most genes were downregulated. Upstream analysis of target enrichment identified GR and NRSF as major regulators of gene changes, and NRSF in particular was primarily enriched among downregulated neuronal genes. Notably, inhibition of NRSF binding to chromatin *via* intracerebroventricular (i.c.v.)-infused decoy oligodeoxynucleotides shortly after early adversity prevented the development of cognitive deficits in LBN rats (Schulmann et al., 2018). Thus, these data implicate NRSF-regulated gene networks in the orchestrated transcriptional programs required for the maturation of spatial memory networks, as well as in the maladaptive silencing of key neuronal genes following early-life adversity.

## Optimal Early-Life Experiences

Important biological phenomena run along a spectrum. If unpredictable maternal care provokes long-term vulnerability, then highly predictable patterns of maternal-derived sensory signals to the developing brain should promote cognitive and emotional resilience long-term. Animal models of optimal, predictable early-life experience, though not as widely studied, include those that apply the handling paradigm advanced by Levine and colleagues (Levine, 1957; Plotsky and Meaney, 1993; Francis and Meaney, 1999; Korosi and Baram, 2009). This involves a brief (15-min) daily separation of rat pups from the mother during the 1st weeks of life. The timing of these bouts of separation is critical, and brief separations will promote increased, predictable sensory input (e.g., licking and grooming) to the pups upon reunion with their mothers (Liu et al., 1997; Fenoglio et al., 2006; Korosi et al., 2010). The recurrent, predictable maternal signals lead to increased resilience to depressive- and anxiety-like behaviors and improved learning and memory (Weaver et al., 2004; Fenoglio et al., 2005, 2006; Champagne, 2008; Korosi and Baram, 2009; Korosi et al., 2010; Singh-Taylor et al., 2018). The mechanisms by which handling improves emotional and cognitive outcomes is not fully understood. Separating pups from their mothers by itself is insufficient. In addition, a single day of handling or repeated but irregular handling are insufficient to promote the molecular and behavioral outcomes (Fenoglio et al., 2006). It thus appears that recurrent and predictable bouts of maternal care, occurring after the brief separations (typically in the same circadian phase), drive the structural and functional brain alterations that promote resilience (Fenoglio et al., 2006; Karsten and Baram, 2013; Davis et al., 2017).

### Circuit Development

The behavioral resilience may result from the influence of optimal early-life experiences on the maturation of stress-related circuits. For example, predictable maternal care results in decreased excitatory synapses onto CRH neurons in the PVN—the exact opposite of the effect of unpredictable maternal care discussed above (Korosi et al., 2010). This decreased synaptic innervation was associated with a diminished frequency of excitatory synaptic currents onto CRH neurons in the PVN, consistent with the observed later-life decrease in stress responsiveness (Korosi et al., 2010; Singh-Taylor et al., 2018). Augmented maternal care further altered the development of other neural systems. For example, the postnatal assembly of preautonomic emotional motor circuits, such as cortical and limbic projections from the bed nucleus of the stria terminalis (BNST), ACe, and visceral cortex to autonomic nuclei, was diminished in animals experiencing neonatal handling (Card et al., 2005). Conversely, in the hippocampus, pyramidal neurons had greater spine density and dendritic complexity, along with enhanced functional synaptic plasticity, in rats that experienced augmented maternal care (Champagne et al., 2008). This result contrasts with the impoverished dendritic trees in rats that experienced fragmented, unpredictable maternal care (Brunson et al., 2005; Ivy et al., 2010; Molet et al., 2016b).

### Epigenetic Alterations

Long-term changes in gene expression may underlie the resilience observed after optimal early-life experiences. For example, in the hippocampus, GR density was increased in adult rats that experienced neonatal handling (Meaney et al., 1985; Liu et al., 1997). In the PVN, a major change induced by augmented, predictable maternal care was decreased CRH expression by P9, prior to the GR change (Plotsky and Meaney, 1993; Liu et al., 1997; Fenoglio et al., 2004, 2005). Mechanistic studies have revealed that reduction of excitatory neurotransmission onto PVN CRH neurons is sufficient to diminish their expression of CRH, among other important neuronal genes related to synaptic function (Singh-Taylor et al., 2018). These alterations in gene expression following optimal early-life experiences can be maintained long-term by epigenetic mechanisms. Large-scale changes in repressive histone modifications of neuronal genes like CRH were initiated by increases in function of the transcriptional repressor, NRSF (Singh-Taylor et al., 2018), the same transcription factor that is implicated in programming LBN-induced cognitive vulnerabilities (Schulmann et al., 2018). These results provide a causal link between neonatal experience, synaptic refinement and induction of epigenetic processes within specific neuronal populations.

## How Does Early-Life Adversity Influence the Maturation of the Brain?

Early-life adversity consists of both physical and emotional components. Physical stress may include hunger or cold, whereas emotional aspects may involve absence or poor care by a stressed parent. In humans, the relative importance of emotional adversity is apparent from comparing outcomes in developing countries, where poverty is almost universal but family structure is stable and nurturing, to outcomes in low-socioeconomic status enclaves in developed countries (Hackman et al., 2010; Crookston et al., 2014; Agidew and Singh, 2018). In contrast, in orphanages where physical wellbeing (i.e., nutrition, shelter) is maintained, but parental care is lacking (and care-giving in general is scarce), emotional and cognitive outcomes of the orphans have been highly deficient (Pollak et al., 2010; Nelson et al., 2011). Both of these scenarios indicate that it is not the physical, but largely emotional, adversity that shapes mental health trajectories.

In our animal model of early-life adversity, we sought to discern the relative roles of physical and emotional adversity in the outcomes discussed above. Physical stress clearly exists in the paradigm (i.e., elevated corticosterone, increased adrenal weights; Brunson et al., 2005; Rice et al., 2008). However, other potential measures of physical stress are absent. For example, cold stress is not prominent: pups do not experience hypothermia in the LBN cages. Core temperatures of control vs. LBN pups are indistinguishable at multiple time points during P2–10 (Figures 1C,D). To consider the possibility that LBN pups might engage their brown adipose tissue (BAT) stores to maintain core temperature and avoid hypothermia, we assessed brown adipose amounts and utilization. The data did not support this hypothesis: a BAT activation test of thermogenesis (Dawkins and Hull, 1964; Himms-Hagen, 1990; Akana and Dallman, 1997) did not reveal any differences between groups, and gross BAT weights did not differ with LBN conditions (Figures 1E,F). This result was not surprising, because the total time the mother spends with her pups is comparable in simulated poverty (LBN) cages and those with a full complement of bedding and nesting material (Molet et al., 2016a). In contrast, the patterns of maternal care are altered, and care is fragmented and unpredictable. It is thus likely that these chaotic maternal sensory signals to the developing brain during a sensitive period provoke the adverse outcomes (Baram et al., 2012; Chen and Baram, 2016; Davis et al., 2017; Walker et al., 2017).

## How Do Optimal Early-Life Experiences Influence Brain Maturation?

Resilience-promoting experiences such as predictable, recurrent maternal sensory signals engage emotion-related circuity. Fos activation of the ACe and BNST, both upstream of PVN CRH neurons, was noted following a single bout of handling and augmented maternal care, but this was insufficient to repress CRH expression in the PVN (Fenoglio et al., 2006). Notably, a week of recurrent daily bouts of augmented maternal care activated the ACe and BNST, as well as the paraventricular nucleus of the thalamus (PVT). The PVT is considered a “stress-memory” center of the brain (Bhatnagar and Dallman, 1998; Bhatnagar et al., 2000). This combined output to PVN CRH neurons in concert with recurrent handling resulted in enduring suppression of CRH expression in these neurons (Fenoglio et al., 2006; Karsten and Baram, 2013). Because neurons that “fire together, wire together,” it is likely that these differential neuronal network activation patterns influence emotion-related circuit development and underlie the long-term resilience resulting from predictable maternal sensory signals.

**Figure 2:**
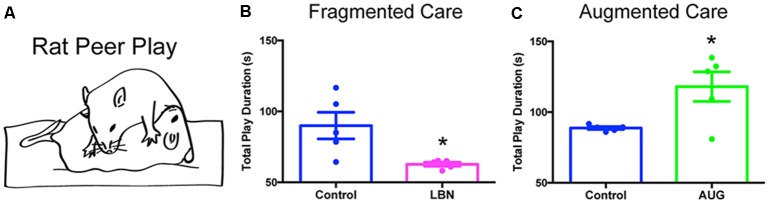
Early-life experiences bidirectionally modulate peer-play behavior. **(A)** Schematic of an example rat peer-play posture, known as pinning. The total play duration graphed in **(B,C)** consists of the summed duration in seconds of all play behaviors, including pinning, pouncing, chasing/following, boxing, and kicking, that a late-adolescent male experimental rat performs, directed toward a juvenile same-sex conspecific, during a 10-min test of social interaction in an open field. **(B)** Fragmented, unpredictable maternal care (LBN) provokes anhedonia, shown here as decreased peer play in a test of social interaction. Data are mean ± SEM; *n* = 5/group; *t*-test with Welch’s correction for unequal variance, *t*_(4.169)_ = 2.879, *p < 0.05. **(C)** Augmented, predictable maternal care (AUG) results in increased peer play in late adolescence. Data are mean ± SEM; *n* = 5/group; *t*-test with Welch’s correction for unequal variance, *t*_(4.070)_ = 2.788, *p < 0.05.

Although less well-explored in this context, reward/pleasure circuits may be engaged by resilience-promoting experiences. Notably, there is evidence that predictable sequences of events activate the reward system (Berns et al., 2001; Rutledge et al., 2014). The dopaminergic reward system is not fully developed until the third postnatal week in rodents (Voorn et al., 1988) and is sensitive to alterations in early-life experiences (Ventura et al., 2013; Peña et al., 2014). Therefore, in analogy to the modulation of visual, auditory, and motor circuit development by visual, auditory, and sensory signals, we speculate that predictable patterns of maternal care provide crucial cues for maturation of the reward/pleasure system. Conversely, unpredictable and fragmented maternal care may result in aberrant maturation of the reward/pleasure circuitry. In support of this notion, LBN has been shown to provoke anhedonia, manifest as reduced sucrose preference, peer play, and cocaine craving in adolescent male rats, accompanied by aberrant connectivity between stress- and reward-related brain regions (Bolton et al., 2018a,b; Figures 2A,B). In turn, preliminary data suggest that predictable, recurrent maternal care results in increased peer play in adolescent male rats (Figure 2C). Thus, patterns of maternal sensory signals may exert bidirectional influence over the maturation of the reward/pleasure system—a hypothesis that warrants further exploration.

## Conclusions

In sum, early-life experiences influence an individual’s risk vs. resilience for emotional and cognitive disorders. The biological processes underlying these profound and enduring effects include influences on circuit development, associated with epigenetic changes. CRH-expressing cells in the neonatal hypothalamus are a key, early target of these experience-dependent mechanisms. Thus, these and other, yet undiscovered, mechanisms by which early-life experiences influence the brain provide tractable targets for intervention.

## Data Availability

All datasets generated for this study are included in the manuscript.

## Ethics Statement

This study was carried out in accordance with the recommendations of the National Institutes of Health. The protocol was approved by the UC Irvine Animal Care and Use Committee.

## Author Contributions

TB designed the experiments. JB, KS, and JD performed the experiments and analyzed the data. TB, JB, and AS wrote the article.

## Conflict of Interest Statement

The authors declare that the research was conducted in the absence of any commercial or financial relationships that could be construed as a potential conflict of interest.
